# Spatial resolution effect of light coupling structures

**DOI:** 10.1038/srep18500

**Published:** 2015-12-18

**Authors:** Juntao Li, Kezheng Li, Christian Schuster, Rongbin Su, Xuehua Wang, Ben-Hur V. Borges, Thomas F. Krauss, Emiliano R. Martins

**Affiliations:** 1 State Key Laboratory of Optoelectronic Materials and Technologies, School of Physics and Engineering, Sun-Yat Sen University, Guangzhou, 510275, China; 2 Department of Physics, University of York, York, YO10 5DD, UK; 3 Electrical Engineering Department, University of São Paulo,Av. Trabalhador Sãocarlense, 400, São Carlos-SP, Brazil

## Abstract

The coupling of light between free space and thin film semiconductors is an essential requirement of modern optoelectronic technology. For monochromatic and single mode devices, high performance grating couplers have been developed that are well understood. For broadband and multimode devices, however, more complex structures, here referred to as “coupling surfaces”, are required, which are often difficult to realise technologically. We identify general design rules based on the Fourier properties of the coupling surface and show how they can be used to determine the spatial resolution required for the coupler’s fabrication. To our knowledge, this question has not been previously addressed, but it is important for the understanding of diffractive nanostructures and their technological realisation. We exemplify our insights with solar cells and UV photodetectors, where high-performance nanostructures that can be realised cost-effectively are essential.

Diffraction gratings and photonic crystals^1^ can be used to achieve efficient coupling in a broad class of devices including integrated photonic circuits^2^,^3^, surface emitting lasers^4^,^5^,^6^, Light Emitting Devices (LEDs)^7^,^8^ and solar cells^9^,^10^,^11^,^12^,^13^,^14^,^15^,^16^,^17^,^18^,^19^. Disordered photonic structures are usually preferred when broad-band coupling is required due to their richer Fourier spectra^18^,^20^,^21^,^22^,^23^,^24^,^25^,^26^,^27^. The disadvantage of disordered structures is that they are difficult to optimise towards specific applications. In order to circumvent this problem, we previously introduced the quasi-random design formalism^23^. Quasi-random structures feature long period unit-cells that are modulated to tailor the Fourier components, resulting in a design that combines the rich Fourier spectra associated with disordered structures with the high degree of control associated with periodicity. We also showed that broad-band (covering the entire visible part of the spectrum) and highly efficient coupling of light into waveguides can be achieved using such quasi-random nanostructures^23^. Broadband coupling is particularly important in the context of light trapping in thin-film photovoltaics^15^. Similarly, the characteristics of light trapping are also important for the inverse problem of extracting light from LEDs and for high performance thin film photodetectors.

In spite of the importance of broad-band light coupling and light trapping in current state-of-the-art technologies, there have only been a few investigations into the physics behind such processes. Among the most insightful contributions are the analytical models developed to estimate the limits of light trapping performance^11^,^28^. The disadvantage of these models, however, is the oversimplification required for their description. Furthermore, even though the physics of broad-band light trapping is intimately related to the Fourier characteristics of the coupling surface, few studies have attempted to elucidate and characterize this relation^29^. Such studies are particularly important because knowledge of the desired Fourier properties greatly facilitates the design of the coupler and the choice of fabrication technology. For example, it has been shown that amorphous structures – which involve very low-cost fabrication – feature Fourier spectra that are much more suitable for the problem of light trapping than conventional photonic crystal couplers^23^. A related problem that has largely been ignored is the spatial resolution required for fabricating the corresponding nanostructures. This problem is reminiscent of the Heisenberg uncertainty principle: broad-band coupling requires structures that feature large grating vectors and provide a sizeable momentum, which in turn results in small spatial features. Knowledge of the highest spatial frequency (or equivalently minimum feature size) required is therefore fundamental for informing fabrication constraints and costs.

Here, we identify a general design principle based on the Fourier properties of the coupling surface and investigate the impact of spatial resolution on the performance. We find that, once the optimum Fourier properties are identified, the performance becomes very tolerant against fabrication imperfections. Importantly, these findings apply not only to the structures investigated in this paper, but also to any structure that has similar Fourier properties. Our approach takes advantage of the unprecedented level of control over the Fourier spectrum afforded by quasi-random nanostructures. We first investigate the dependency of broad-band light coupling on the Fourier spectrum of the coupling surface and show how broad-band coupling is a consequence of Fourier energy concentration in specific bands. Second, we investigate the impact of real-space resolution limitations on the light coupling performance. We find that the highest resolution significantly depends on the available mode spectrum and that the technologically required resolution is lower than the resolution one may naively assume. We illustrate these observations using two absorbing systems: a silicon thin film relevant for solar cells, and a thin layer of gallium nitride (GaN), which features strong absorption and is employed in ultra-violet (UV) photodetectors. We find that a near-optimum coupling performance is already achieved for spatial resolutions higher than 20 μm^−1^ and 30 μm^−1^ for the silicon and GaN systems, respectively, typically twice as low as one would naively assume. These results are a useful guide for determining the trade-off between performance and fabrication requirements.

## Results

**Figure 1:**
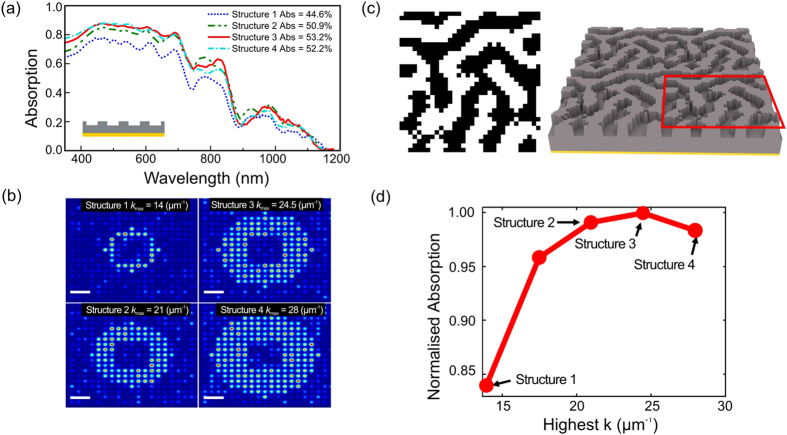
Dependence of light trapping performance on the Fourier properties of the coupling surface. (**a**) Absorption spectrum of 4 different coupling surfaces. Inset shows the cross-section of the investigated 500 nm thick silicon thin film sitting on a perfect mirror. (**b**) Fourier distribution of the corresponding structures. The white bar is Δk = 10 μm^−1^ long. As the energy ring in *k*-space is broadened by adding more spatial frequencies to the coupler (illustrated by the evolution from structure 1 to structure 4) the thin-film absorption is enhanced towards the short-wavelength spectral region (λ < 800 nm). The integrated absorption is optimum when the coupling surface concentrates its Fourier energy in the region 10.5 μm^−1^ < k < 24.5 μm^−1^ (structure 3). (**c**) Example of a unit-cell of the coupling surface. The unit-cells are 1.8 μm long. (**d**) Integrated absorption as a function of the highest spatial frequency. The absorption shows a rapid increase up to k ~ 20 μm^−1^. This is the point where the entire absorption spectrum can be accessed by the coupling surface. Beyond this point, the absorption is only marginally increased and is reduced for frequencies higher than 25 μm^−1^ (notice that the structure corresponding to the point at k = 17 μm^−1^ is not shown in (**a,b**) to avoid overloading these figures).

The most important parameter of a coupling surface is its period. Changing the period is equivalent to changing the location of the Fourier components in *k* - space. Since *k* - space defines the operational frequency window, the band of optimum coupling moves across the spectrum as the period is changed. The second parameter is the range of *k* - space covered by the coupling surface. For example, limited light trapping performance achieved by singly periodic structures is due to the fact that they only cover a narrow range in *k* - space. This narrow range explains their limited performance in photovoltaics because band- limited coupling results in band-limited absorption of sunlight. This limitation can be overcome by adding more periods to the nanostructure, thereby adding Fourier components. The benefit of adding Fourier components can be seen in Fig. 1a, which shows the absorption spectra of a 500 nm thick silicon slab patterned with a range of quasi-random structures, with the corresponding Fourier spectra in Fig. 1b. All designs were obtained using a square lattice with a 1.8 μm long unit-cell containing 32 × 32 pixels, thus resulting in a pixel size of 56 nm. An example of such a unit-cell is shown in Fig. 1c. Structure 1–4 are designed such that their lowest Fourier components are located at k = 10.5 μm^−1^, which corresponds to a period of Λ = 2π/k = 600 nm. The light trapping mechanism is based on the excitation of waveguide modes of the structure. These modes refer to the electromagnetic distribution inside the waveguide and correspond to solutions of Maxwell’s equations that are oscillating inside the waveguide and evanescent outside it. Notice that the diffractive structures, which are responsible for the excitation of these modes, also out-couple the modes from the waveguide. In this way, these waveguide modes become leaky in the presence of the diffractive structures and are therefore called quasi-guided modes. Since the excitation of quasi-guided modes requires sub-wavelength periods, the component at k = 10.5 μm^−1^ provides the strongest coupling in the wavelength region λ > 600 nm. As we add higher momentum components progressively from structure 1 to structure 4, we can increasingly access the shorter wavelength (λ < 800 nm) range. As the Fourier space is broadened, the absorption is enhanced in the shorter-wavelength region with no detriment in the longer wavelength range. Notice that the absorption enhancement is optimal for structure 3, and that the addition of further Fourier components in structure 4 reduces the overall absorption. The highest spatial frequency (Fourier component) of structure 3 is k = 24.5 μm^−1^, which corresponds to a period of Λ = 260 nm. Therefore, optimum broad-band coupling for a thin silicon film is achieved when the Fourier energy is concentrated between the periods of Λ = 260 nm (k = 24.5 μm^−1^) and Λ = 600 nm (k = 10.5 μm^−1^). In order to quantify the absorption enhancement, the normalised integrated absorption calculated assuming AM1.5G spectrum for sun light is shown in Fig. 1d as a function of the highest spatial frequency (the integrated absorption is also shown as the legend in Fig. 1a). As expected, the integrated absorption increases as the spectrum is broadened (spatial frequency is increased). Notice, however, that the enhancement reaches a plateau around 20 μm^−1^. Beyond this point, the absorption is only marginally increased up to the optimum point at 24.5 μm^−1^ and then decreases for even higher spatial frequencies.

In order to better understand the origin of this optimum Fourier region, we start with the intuitive assumption that the periodic diffractive structure needs to couple to all available guided modes of the thin film for optimum performance. The *k*-vectors of these guided modes, ignoring dispersion, relate to the corresponding wavelengths as follows:


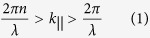


where *n* is the refractive index of the waveguide, which we assume to be silicon. Equation (1) can be re-expressed as


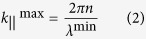


where *k*_||_^max^ is the highest required spatial frequency and *λ*^min^ is the smallest wavelength we want to couple into the film. Similarly, the lowest spatial frequency can be defined as:


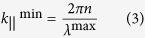


**Figure 2:**
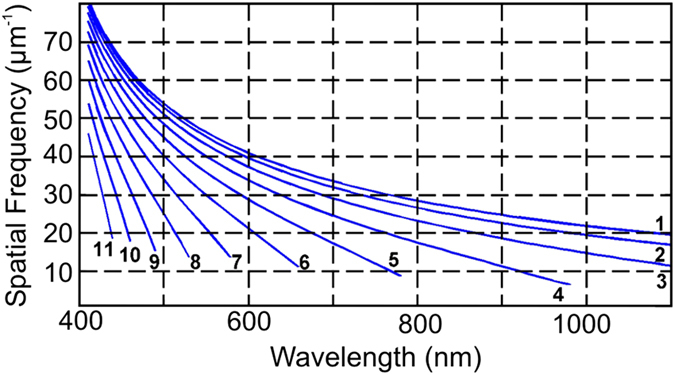
Modal dispersion of a 500 nm thick silicon planar waveguide on top of a perfect metal. The corresponding mode number is indicated on each curve. If the spatial frequency is too low, only a few modes can be coupled inside the absorption band of silicon. For example, only modes 4 and 5 can be coupled for a spatial resolution of 10 μm^−1^. As the spatial frequency of 20 μm^−1^ can already access the entire absorption band, the absorption saturates for higher spatial frequencies.

Assuming *n* = 3.5 for silicon and a wavelength window of interest between 400 and 1100 nm, this simplistic analysis results in *k*_||_^max^ = 55 μm^−1^ and *k*_||_^min^ = 5.7 μm^−1^, which corresponds to a minimum period of 115 nm and a maximum period of 1100 nm, respectively. As we can see from Fig. 1a,d, these values do not correspond to the optimum values of *k*_||_^max^ = 24.5 μm^−1^ and *k*_||_^min^ = 10.5 μm^−1^ we found previously. The reason for this discrepancy can be understood via the modal dispersion of the waveguide, which is shown in Fig. 2 for a 500 nm thick silicon slab waveguide. As is apparent from this figure, a spatial frequency of 5 μm^−1^ cannot excite any mode in the wavelength region below λ = 1100 nm, where silicon absorbs, because all the modes are below cut-off, with cut-off being indicated by the fact that the modal dispersion curves do not extend all the way to *k* = 0. A spatial frequency of 10 μm^−1^, however, can excite two modes (modes 4 and 5).

Notice that, even though the lowest order modes (modes 1–3) can be excited by low spatial frequencies (*k* < 10 μm^−1^), the excitation occurs in the wavelength region where silicon does not absorb (*λ* > 1100 nm), so is not relevant for the application. Conversely, the highest order modes (modes > 5) cannot be excited by *k* = 10 μm^−1^ because they are still below cut-off, i.e. the dispersion curves for these modes do not reach the *k* = 10 μm^−1^ line. The *k* = 10 μm^−1^ line therefore sets the lower bound of useful spatial frequencies. How about the upper bound? As *k* increases, more and more modes are coupled, such that by *k* ≈ 20 μm^−1^ the coupling surface can address all 11 available modes, thus covering the entire absorption band. Correspondingly, this is where the saturation regime begins. Beyond this point, the benefit of adding further Fourier components is limited and, in fact, can be even detrimental. Indeed, higher spatial frequencies cannot couple in the long wavelength region. For example, the spatial frequency at 30 μm^−1^ can only couple to wavelengths shorter than 750 nm. As the presence of these higher spatial frequencies reduces the relative contribution of the lowest spatial frequencies, which usefully couple to quasi-guided modes, their overall effect is detrimental. These observations explain why the performance saturates for a *k*_||_^max^ ≈ 20 μm^−1^ instead of the *k*_||_^max^ = 55 μm^−1^ predicted by the simplistic analysis of equ. 2. Inspection of the waveguide dispersion can, therefore, help to define the spatial frequencies needed to design a given coupler performance.

Once the optimum Fourier distribution has been identified, the next question is the fidelity with which the structures need to be reproduced. The quasi-random nanostructures used so far were defined by a matrix of 32 × 32 pixels in real space. For a period of 1800 nm, this matrix size corresponds to a highest spatial frequency of 56 μm^−1^ (minimum feature size of 1800/32 = 56 nm, corresponding to a smallest period of 112 nm). Notice that the highest spatial frequency here is defined as


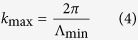


where Λ_min_ is the smallest period (corresponding to twice the length of the smallest feature size). This definition is different from the highest spatial frequency of Fig. 1d, which is taken directly from the Fourier transform.

**Figure 3:**
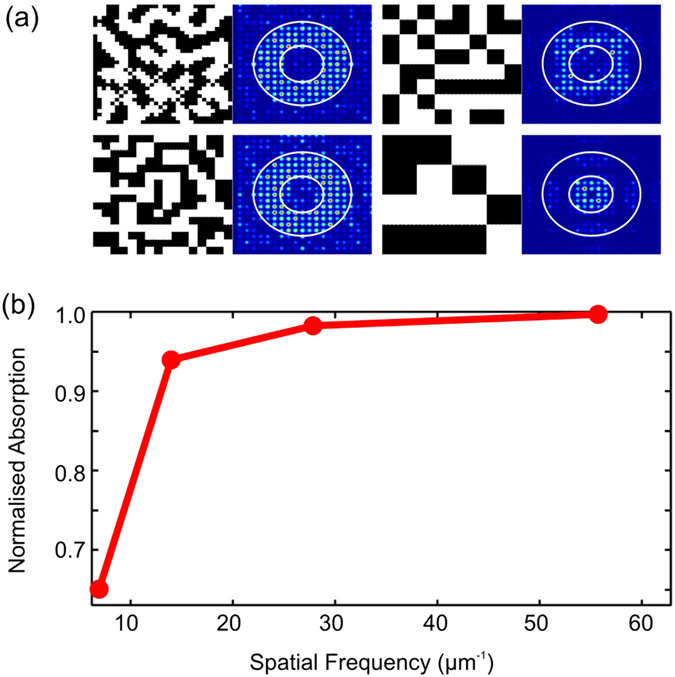
Structures and absorption with different spatial resolution. (**a**) Real and Fourier space of structures with different spatial resolution. The boundaries of the target region in Fourier space are highlighted by the circles. The top-left structure has a resolution of 56 nm; bottom-left structure has resolution of 112 nm; top-right structure has resolution of 225 nm and bottom-right structure has resolution of 450 nm. All unit-cells are 1.8 μm long. (**b**) Absorption dependence on spatial frequency. The absorption quickly saturates for structures with spatial frequencies above 20 μm^−1^.

In order to investigate how far the features size can be relaxed, we designed patterns with fewer pixels and Fourier distributions as close as possible to the optimum Fourier distribution of design 3 in Fig. 1b, then reduced the number of pixels used to make up the supercell. The structures investigated (see Fig. 3a) were defined with matrix resolutions of 32 × 32 (top-left), 16 × 16 (bottom-left), 8 × 8 (top right) and 4 × 4 (bottom-right), corresponding to the highest spatial frequencies of 56, 28, 15 and 7 μm^−1^, respectively (minimum corresponding feature sizes of 56 nm, 112 nm, 225 nm and 450 nm).

In Fig. 3a, the boundaries of the target range in Fourier space are highlighted by circles. Interestingly, a very well defined ring can already be achieved with a highest spatial frequency of 28 μm^−1^ (16 × 16 pixels). A more blurred spectrum, but in which we can still distinguish the energy concentration in the desired region with still relatively good performance, is observed for the structure with highest spatial frequency of 15 μm^−1^ (8 × 8 pixels), while the performance drops significantly for the 4 × 4 pixel structure. Figure 3b shows that the absorption increases monotonically with the spatial frequency up to a saturation point. Defining saturation as the point where the performance drops by ~5% of the maximum, we identify that saturation occurs around *k* = 20 μm^−1^, which is very similar to the study shown in Fig. 1d. Notice, however, that differently from Fig. 1d, the performance does not fall for higher frequencies. This difference arises because the higher spatial frequencies of the designs of Fig. 3a do not necessarily result in large Fourier components. Instead, high spatial frequencies correspond to better spatial resolution, thus contributing to a more accurate reproduction of the desired Fourier spectrum, Very similar results were obtained with quasi-random structures with 3.6 μm period designed to have the Fourier energy concentrated in the same region (see Supplementary Information). These results confirm that the performance is dependent on the general Fourier properties, and not on the detailed spatial distribution.

**Figure 4:**
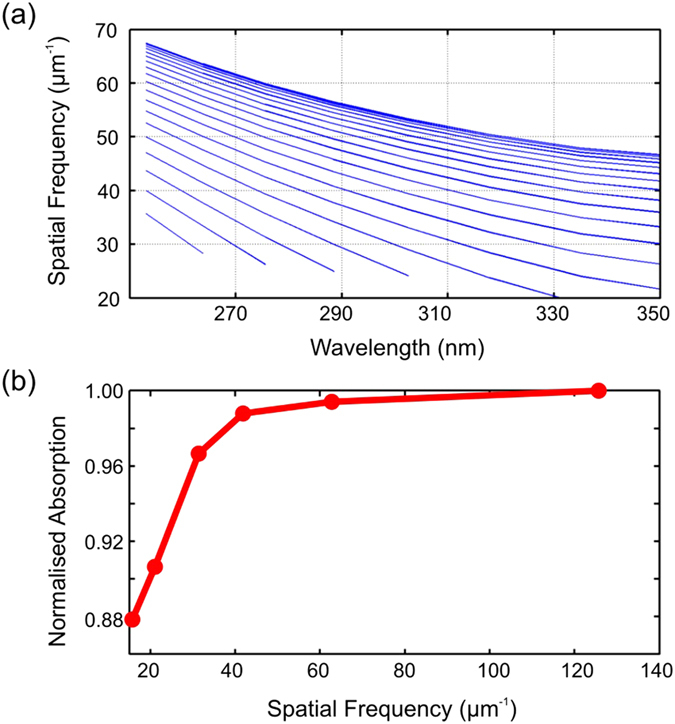
(**a**) Modal dispersion for a 1 μm thick GaN planar waveguide. (**b**) Absorption as a function of spatial resolution for the GaN waveguide. The absorption profile saturates for spatial resolution higher than ~30 μm^−1^ because this is the point that can access the entire absorption band.

In order to verify the generality of our method, we carried out the same study as shown in Fig. 3 for a different absorbing system, namely a 1 μm thick GaN film that represents a high-performance UV detector with a target operation range in the near-UV (210 nm < λ < 350 nm). Notice that, due to the different absorption characteristics, (see Supplementary Information), the structures and results obtained in Fig. 3 cannot simply be carried over to the GaN structure by scaling the parameters with respect to the wavelength in the material. Figure 4a shows the dispersion band of the GaN waveguide and Fig. 4b shows the absorption as a function of spatial resolution (maximum spatial frequency). This time, the waveguide features more modes due to the increased thickness. As is apparent from the dispersion diagram, a minimum spatial resolution around 30 μm^−1^ is necessary to cover the entire absorbing region up to λ~ 350 nm. Again, this is also the point where saturation is reached. Therefore, the conclusion reached for silicon is also confirmed by the GaN film: saturation is reached for the spatial frequency that covers the entire absorption spectrum. Furthermore, the absorption is expected to decay quickly for spatial frequencies below 30 μm^−1^, as can indeed be observed in Fig. 4b.

In order to experimentally confirm the above insights, we fabricated a range of structures by e-beam lithography on a 500 nm silicon film placed on a glass substrate.

**Figure 5:**
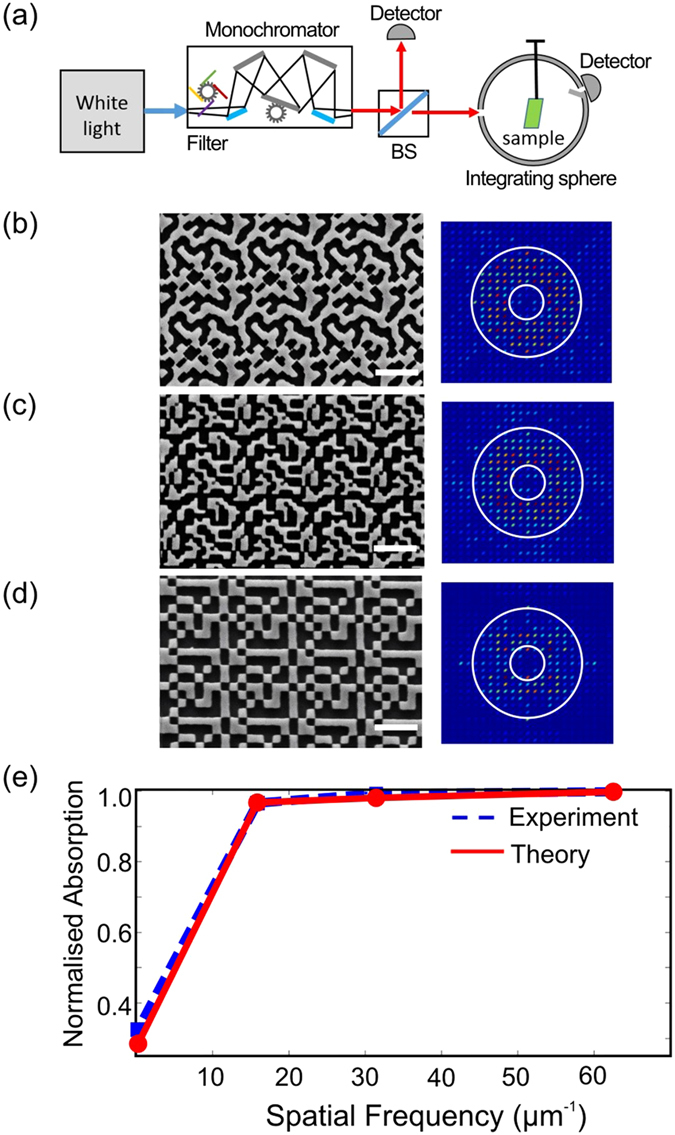
Experiment results. (**a**) Experimental set-up for absorption measurement in the patterned structures. An integrated sphere is used to collect all diffraction orders. (**b–d**) left: SEM micrographs of fabricated structures with spatial resolutions of (**b**) 63 μm^−1^, (**c**) 31 μm^−1^ and (**d**) 15 μm^−1^; right: corresponding Fourier spectra (calculated from the SEM micrographs). The white bar is 1 μm long (**e**) Comparison between measured and calculated absorption profiles.

The real and the Fourier distributions of the fabricated structures are shown in Fig. 5b–d. The geometries in Fig. 5b–d correspond to the geometries of Fig. 3a top left, bottom left and top right, respectively. The Fourier transform was calculated directly from the Scanning Electron Microscope (SEM) micrographs. Figure 5e shows a comparison between the integrated absorption for the structures with different spatial resolution. As is apparent from this plot, the same tendency observed from theory is confirmed by experiment and the “knee” of the curve separating the region of saturation from the region of quick decay again lies around a spatial frequency of 20 μm^−1^.

## Discussion

The dependence of the light trapping performance of a thin film semiconductor absorber on the Fourier properties of a coupling surface has been identified. It was found that the highest required spatial frequency is given by the modal dispersion of the waveguide: the highest spatial frequency corresponds to the point for which the entire absorbing region can be accessed. This requirement does not depend on the details of the coupling surface and can be used as the criteria to design and implement coupling surfaces of any kind. We have approached the spatial resolution issue from two different directions, namely a) via the harmonics we include into the design (Fig. 1a,b,d) and b) via the smallest spatial features present in the pattern (Figs 3a,b and 4b). Remarkably, both approaches yield very similar results. This agreement highlights the fact that the spatial frequency content of the coupler is the dominant parameter that determines its performance. The fact that the two approaches are not identical, however, is highlighted by the discrepancy apparent at higher spatial frequencies. In Fig. 1d, the performance drops slightly as further harmonics are added, while in Fig. 3b the performance tends towards unity as more spatial frequencies are added. This discrepancy arises because when higher spatial frequencies are added to the design (Fig. 1b), these higher frequencies are not needed for improved performance but, instead, they dilute the performance of the coupler. In Fig. 3b, however, the presence of higher spatial frequencies corresponds to the fact that the pattern is reproduced with higher accuracy. Even though we describe both cases with a spatial frequency in units of μm^−1^, the physical meaning is different. Our key insight is that the optimum Fourier region required for optimum broadband coupler operation depends on the modal dispersion of the waveguide and is substantially smaller than the region expected from a simplistic analysis. Applied to the problem of light trapping in thin film silicon solar cells, this insight highlights that optimum light trapping requires a real space resolution around 20 μm^−1^, and benefits only marginally from smaller feature size.

## Methods

### Quasi-random structure design

The quasi-random structure was designed by a direct binary search algorithm. The details can be found in reference 23.

### Silicon thin film fabrication

The silicon thin films were lifted off from a silicon on insulator (SOI) wafer with a 500 nm thick silicon layer^30^. In order to remove the substrate, the SOI was bonded to a glass substrate with benzocyclobutene (BCB), followed by annealing at 250 °C for 1 hour to solidify the BCB. The backside silicon substrate was then polished and reduced to a thickness of 100 μm. The remaining silicon was chemically etched with potassium hydroxide (KOH) solution at 80 °C, and finally, the 2 μm oxide layer was etched away in a 10% solution of hydrofluoric acid (HF).

### Silicon thin film patterning

The ebeam resist AR-P 6200.9 (ALLRESIST GMBH) was spin-coated on the silicon film and baked at 180  °C for 5 minutes. The ebeam resist was subsequently patterned using a Raith Voyager ebeam system, followed by resist development in Xylene at 19.7 °C for 2 minutes and dry etching with a CHF3 and SF6 gas mixture (14.5:12.5) to transfer the pattern from the resist into the silicon layer.

### Absorption measurement

The patterned thin film absorption was measured in the set-up of Fig. 5a, which is comprised of an integrating sphere, a white light source (LEUKOS SM 30) attached to a monochromator (Zolix, Omni λ 1509) equipped with a diffraction grating (Richardson, 1200 lines/mm & blazed at λ = 600 nm) and a long pass filter. The total transmission and total reflection were measured using a PIN femtowatt silicon detector (Thorlabs, PDF10A) and the background signal was obtained by keeping the sample inside the sphere without direct interaction with the laser. In addition, any fluctuations of the laser where taken into account by a reference detector (Thorlabs, PDA100A). Then the signals were recorded by two digital multimeters (Keithley, 2110 DMM) for each wavelength. Finally, the absorption measurements were normalized to a glass cover slide. Notice that there was no back-side metal on the sample.

## Additional Information

**How to cite this article**: Li, J. *et al.* Spatial resolution effect of light coupling structures. *Sci. Rep.* **5**, 18500; doi: 10.1038/srep18500 (2015).

## Supplementary Material

Supplementary Information
