# Supplementary material for: Spatial resolution effect of light coupling structures
Source: Sci Rep. 2015 Dec 18;5:18500. doi: 10.1038/srep18500 (PMC4683437; doi:10.1038/srep18500)
Supplement: Supplementary Information [file srep18500-s1.pdf]

SUPPLEMENTARY INFORMATION

Spatial resolution effect of light coupling structures

JUNTAO LI* ,[1] KEZHENG LI,[1,2] CHRISTIAN SCHUSTER[2], RONGBIN SU[1], XUEHUA WANG,[1] BEN-HUR V. BORGES,[3] THOMAS F. KRAUSS[1,2] AND EMILIANO R MARTINS[3,4]

[1]*State Key Laboratory of Optoelectronic Materials and Technologies, School of Physics and Engineering, Sun-Yat Sen University, Guangzhou, 510275, China*

[2]*Department of Physics, University of York, York, YO10 5DD, UK*

[3]Electrical Engineering Department, University of São Paulo,Av. *Trabalhador Sãocarlense, 400, São Carlos-SP, Brazil*

[4]*Departamento de Física, Universidade Federal de São Carlos, 13565-905 São Carlos, São Paulo, Brazil*

**Corresponding author: lijt3@mail.sysu.edu.cn*

S1 – Spatial resolution for a 3.6 µm period

The study shown in figure 4 was also carried out for a period of 3.6 µm in order to verify that our conclusions hold generally and are determined by the Fourier components of the coupling surface and not by its specific spatial structure. The structures were designed to concentrate the Fourier energy in the region 10 $\mu m^{-1} < k < 25\ \mu m^{-1}$, identical to the 1.8 µm period case. Notice, however, that the larger period results in a smaller spacing between neighbouring harmonics: whereas for a period of 1.8 µm the spacing is $\Delta k = 3.5\ \mu m^{-1}$, for a period of 3.6 µm the spacing is $\Delta k = 1.75$

μm^{-1}. Therefore, the same region in Fourier space contains twice as many harmonics for the structure with period of 3.6 μm (figure S1) than the structure with period of 1.8 μm (figure 4).

The real space and their corresponding Fourier representation of four different designs with a period of 3.6 μm are shown in Figure S1a. The targeted Fourier region is highlighted by white circles. The designs were obtained using a 64 x 64 matrix (top left), a 32 x 32 matrix (bottom left), a 16 x 16 matrix (top right) and a 8 x 8 matrix (bottom right). The highest spatial frequencies of these designs are, respectively, 56 μm^{-1}, 28 μm^{-1}, 14 μm^{-1} and 7 μm^{-1}. Inspection of Figure S1a shows that the Fourier spectra of the structures with resolution of 56 μm^{-1} and 28 μm^{-1} are very similar, so the corresponding absorption is also similar, as can be seen in Figure S1b. The Fourier spectrum of the structure with resolution of 14 μm^{-1}, on the other hand, is more spread out of the optimum region, so the integrated absorption is reduced as compared to the highest resolution design. The spectrum of the structure with resolution of 7 μm^{-1}, however, is nearly completely outside the optimum region, thus resulting in a more pronounced reduction of absorption. This comparison shows that the same range of spatial frequencies is required for best coupling, irrespective of the period used.

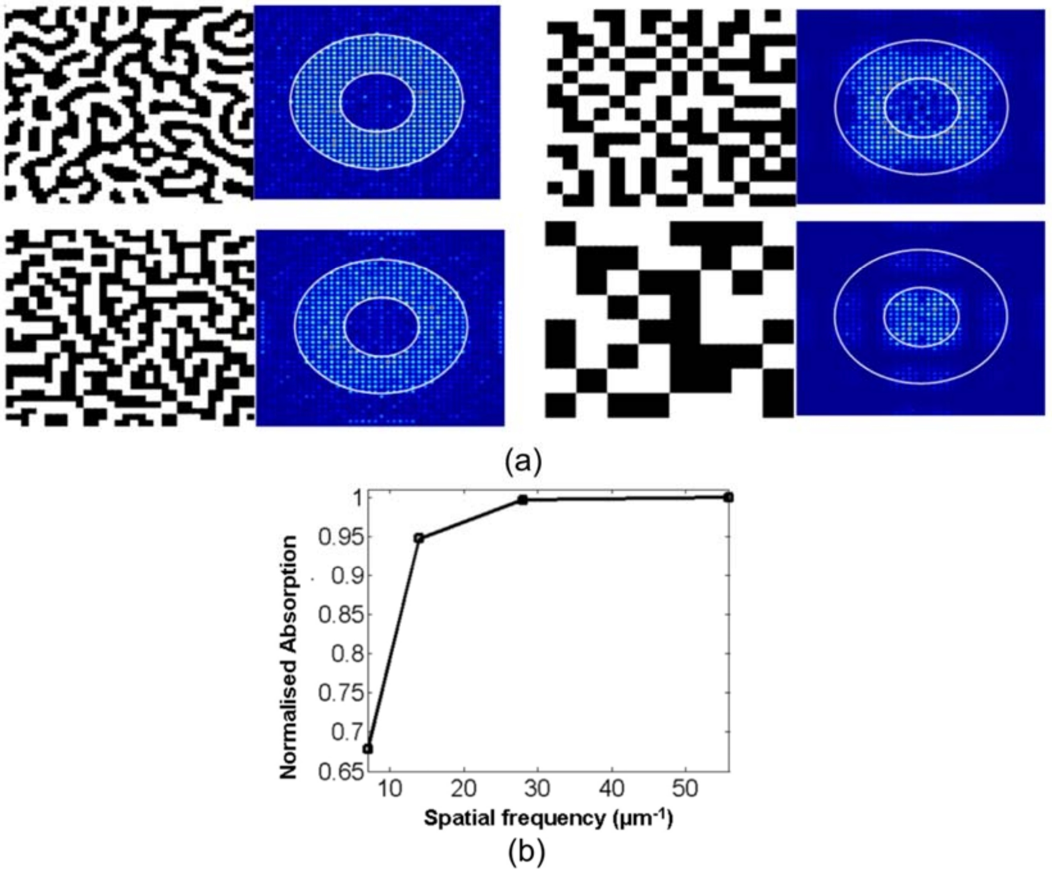

Figure S1 a) Real space and Fourier distribution of four designs using a period of 3.6 µm. The top left figure has a resolution of 56 µm-1, the bottom left figure has a resolution of 28 µm-1, the top right figure has a resolution of 14 and the bottom right figure has a resolution of 7 µm-1. b) Integrated absorption as a function of spatial frequency.

S2 – Refractive index and absorption for silicon and GaN

The absorption of the Silicon and GaN structures were calculated using the refractive index data from [S1] and [S2], respectively. For the waveguide modes, only the real part of the refractive indexes was used. The real part of the refractive index is shown in Figure S2a and the absorption coefficient is shown in Figure S2b.

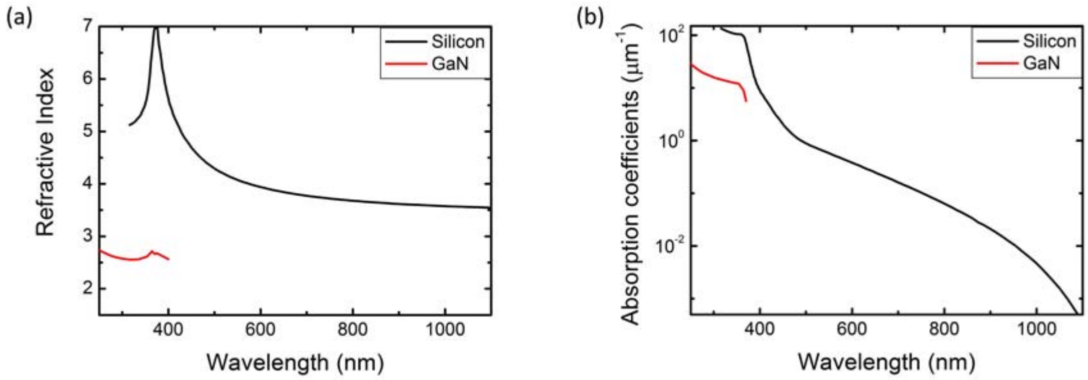

Figure S2 a) Refractive index (real part) of silicon and GaN, taken from [S1] and [S2], respectively. Absorption coefficients of silicon [S1] and GaN [S2].

S3 – References

S1: E.D. Palik, *Handbook of Optical Constants of Solids* (Academic, Orlando 1985)

S2: S. Adachi, *Optical constants of crystalline and amorphous semiconductors: Numerical data and graphical information* (Kluwer Acadmeic Publisher)
